# *Beauveria bassiana*: a new *N*^6^-(2-hydroxyethyl)-adenosine–producing fungus

**DOI:** 10.1080/21501203.2017.1375040

**Published:** 2017-09-11

**Authors:** Kuanbo Liu, Fen Wang, Wenzhao Wang, Caihong Dong

**Affiliations:** a Guizhou Provincial Key Laboratory of Fermentation Engineering and Biological Pharmacy, Guizhou University, Guiyang, China; b School of Liquor-making and Food Engineering, Guizhou University, Guiyang, China; c State Key Laboratory of Mycology, Institute of Microbiology, Chinese Academy of Sciences, Beijing, China

**Keywords:** Nucleosides, *N*-(2-hydroxyethyl)-adenosine, *Beauveria bassiana*, *Cordyceps*

## Abstract

*N*^6^-(2-Hydroxyethyl)-adenosine (HEA), which was the first calcium antagonist derived from biological sources, has been intensively investigated because of its ability to inhibit tumour cell proliferation, restrain inflammation, protect kidneys and function as a sedative and insecticide. Up to now, the production of HEA has been detected only in a few species such as *Cordyceps pruinosa, C. militaris* and *Isaria tenuipes*. Here, we found a new HEA-producing fungus, which was identified as *Beauveria bassiana* based on morphological and phylogenetic characteristics. The HEA production was verified by reversed-phase high-performance liquid chromatography/mass spectrometry in this fungus. Moreover, HEA was also detected in the mycelia of two other *B. bassiana* strains from different origins, but not in the culture medium of all tested *B. bassiana* strains. The maximum production of HEA (0.8483 ± 0.0439 mg/g mycelia dry weight) was achieved on day 7. The fungal biomass and kinetics of HEA production exhibited similar trends over the duration of the culture period. This is the first report describing HEA production in *B. bassiana*, which suggests that this fungal strain may have new applications as a source of HEA.

## Introduction

Nucleosides are important for regulating diverse physiological processes in human and for preventing or treating diseases (Hu and Yang 2014). Additionally, nucleosides, including adenosine, cordycepin and *N*^6^-(2-hydroxyethyl)-adenosine (HEA), represent the major active components in *Cordyceps sensu lato* (Gong et al. 2004; Li et al. 2006). HEA was the first calcium antagonist derived from biological sources, and can be used as an inotropic agent (Furuya et al. 1983). Recent studies have revealed HEA has various biological activities. For example, it can inhibit the proliferation of tumour cells *in vitro*, including Lewis lung cancer and K562 erythroleukemia cells (Lu et al. 2013; Zhu et al. 2013). HEA regulates cerebral and coronary circulation and appears to function as a sedative in pharmacological tests (Wang et al. 2013). The mechanism underlying the analgesic activity of HEA differs from opioids, a widely used drug for analgesic. More importantly, HEA is neither addictive nor affected by pepsin, which is the advantage over opioids (Chai et al. 2004). Peng et al. (2015) reported that HEA protected mice from renal ischemia reperfusion injury. Studies confirmed that HEA and human serum albumin interacted to form a complex by hydrophobic interaction (Cui et al. 2007; Meng et al. 2015b). HEA also attenuated lipopolysaccharide-induced pro-inflammatory responses by suppressing the toll-like receptor 4-mediated nuclear factor-κB signalling pathway (Lu et al. 2015). A recent study revealed that HEA exhibited insecticidal activity against *Plutella xylostella* larvae by targeting an adenosine receptor (Fang et al. 2016), suggesting an environmentally friendly pesticide (Chai et al. 2015).

HEA was firstly isolated from *Cordyceps* species. Furuya et al. (1983) tested for the presence of Ca^2+^ antagonists and inotropic agents in the water–ethanol extracts from mycelia of 17 *Cordyceps* species and 6 *Isaria* species using the left atrium of a guinea pig heart in an *in vitro* system. The mycelial extracts from two *Cordyceps* species (i.e. *C. pruinosa* and *C. coccidiiocola*) and four *Isaria* species (i.e. *I. japonica* and three incertae sedis species) were highly active for inotropic. This active substance was identified as HEA. The production of HEA by *C. pruinosa* mycelia in static liquid and solid-state cultures was verified by high-performance liquid chromatography/mass spectrometry (HPLC-MS) (Meng et al. 2014).

HEA has also been identified in several *Cordyceps sensu lato* species, such as *I. cicadae, C. militaris* and *I. tenuipes*. Content of HEA in *I. cicadae* mycelia cultured in barley was higher than those cultured in other substrates (Ke and Lee 2016). *C. militaris* mycelia and fruiting bodies produce HEA (Zhu et al. 2013; Zhang et al. 2016), as does *I. tenuipes* grown under submerged culture conditions (Liu et al. 2012; Lei et al. 2014). It was reported that the amount of HEA in *Ophiocordyceps sinensis* (Berk.) G. H. Sung, J. M. Sung, Hywel-Jones & Spatafora [≡ *C. sinensis* (Berk.) Sacc.] is a reliable indicator of the overall health benefits of the medicinal fungus (Phillip et al. 2008). However, HEA production in the stroma and submerged mycelia of *O. sinensis* has not been detected (Liu et al. 2017).

About 538 *Cordyceps sensu lato* species have been identified by now (http://www.indexfungorum.org/names/Names.asp, 10 July 2017). However, HEA has been detected in only a few species such as *C. pruinosa, C. militaris* and *I. tenuipes*. Consequently, identifying new sources of HEA from *Cordyceps sensu lato* species is warranted (Liu et al. 2017). In this study, we detected HEA in the entomopathogenic fungi collected in our laboratory and found a new HEA-producing fungus, which was identified as *Beauveria bassiana*. We monitored the HEA production in a liquid culture of *B. bassiana*. This is the first report of *B. bassiana* producing HEA, suggesting a new source of HEA.

## Materials and methods

### Fungal strains and culture conditions

More than 20 entomogenous fungal strains collected in our laboratory were used to screen HEA-producing fungi. These strains were maintained on potato dextrose agar (PDA) at 4°C. They were inoculated in the same medium in Petri dishes and incubated at 20°C or 25°C for 7–15 days. Liquid cultures were subsequently prepared by inoculating 80 mL potato dextrose broth (PDB) in 250-mL Erlenmeyer flasks with 5-mm mycelial plugs. The flasks were incubated at 20°C or 25°C with shaking (150 rpm) for 7–15 days.

#### Screening for HEA-producing fungi

Samples were extracted by deionised water using the ultrasonic extraction method for 0.5 h and filtered through a 0.45-mm filter membrane prior to injection into the HPLC system.

The production of HEA was analysed by reversed-phase HPLC using a Waters 2695 Separations Module (Waters Corporation, Milford, CT, USA) equipped with a built-in quaternary pump, a 120 autosampler, the Waters 2998 Photodiode Array Detector and the Empower program for analysing data. A pre-packed Puritex C18 column (4.6 × 250 mm, 5 μm particle size; Beijing Greenherbs Science and Technology Development Co. Ltd., Beijing, China) was used. Samples were eluted in the mobile phase, which consisted of acetonitrile: double-distilled H_2_O (0.1% acetic acid) (5:95), for 20 min at 25°C. The flow rate was 1 mL/min and the injection volume was 10 μL. Nucleosides were monitored and quantified at 260 nm. Commercially available adenosine (Sigma, Munich, Germany) and HEA (Shanghai PureOne Biotechnology, Pudong, Shanghai) were dissolved in distilled water and used as calibrators.

### Verification of HEA production

The production of HEA was verified by HPLC-MS. An Agilent 6520 Accurate-Mass Quadrupole Time-of-Flight mass spectrometer (Santa Clara, CA, USA) was equipped with an electrospray ionisation interface. Peaks were detected in the positive ionisation mode. The HPLC-MS conditions were as follows: capillary voltage, 3500 V; drying gas temperature, 300°C; drying gas, 11.0 L/min; fragmentor voltage, 130 V; and nebuliser pressure, 30 psi. Data were collected on an HP ChemStation. Spectra were scanned over the mass range 80–2000 *m*/*z* at 1.03 spectra/s. The HEA standard was dissolved in distilled water and used as a calibrator.

### Identification of an HEA-producing strain

Strains were identified based on morphological characteristics and phylogenetic analysis. The HEA-producing fungal strain 351 was grown on malt extract agar (MEA; 20 g malt extract, 1.0 g peptone, 20 g glucose, 20 g agar and 1 L distilled water) and PDA. Morphological characteristics were examined using a Nikon Eclipse 80i microscope (Nikon Instruments Inc., Tokyo, Japan) and a preliminary identification was made based on fungal colony morphology and spore characteristics.

For strain identification, genomic DNA was prepared with the cetyltrimethylammonium bromide method (Doyle and Doyle 1987) and the Internal Transcribed Spacer (ITS1-5.8S-ITS2) of the nuclear ribosomal DNA was amplified. The nucleotide sequence has been deposited in GenBank under accession number MF417575 for strain 351.

Phylogenetic analysis based on the ITS sequence was performed to ascertain the identity of the strain. Reference sequences downloaded from GenBank and the sequences obtained in this study were aligned with Clustal X program. Maximum-likelihood analysis was performed using Mega 6.06 with Kimura’s 2-parameter model (Tamura et al. 2013). Bootstrap analyses were conducted with 1000 replicates. Trees were figured in FigTree v1.4.2, with bootstrap values greater than 50% provided. The ITS sequences of *Aspergillus terreus* (GU256759.1) and *A. fumigatus* (KU612366.1) were used as out groups.

### Determination of HEA production by the other *B. bassiana* strains

Two other *B. bassiana* strains were analysed for HEA production, namely CGMCC 3.3575 (from the China General Microbiological Culture Collection Center) and strain 627 (a gift from Prof. Xingzhong Liu, Institute of Microbiology, Chinese Academy of Sciences). These two *B. bassiana* strains were cultured for 7 days at 25°C in enriched PDB medium (200 g potato, 3.0 g peptone, 20 g glucose, 1.0 g KH_2_PO_4_, 0.5 g MgSO_4_ and 1 L distilled water). The mycelia were pelleted by centrifuging the cultures at 4500 rpm for 15 min. The mycelial pellets were washed with distilled water and dried at 45°C to a constant weight, after which the mycelial biomass was determined. The mycelium and culture medium were analysed for HEA content. All experiments were conducted in triplicate.

### HEA production curve during the liquid culture

Twenty-four Erlenmeyer flasks were used to monitor HEA production in liquid cultures. The 250-mL flasks containing 80 mL medium were inoculated with a seed culture (10% volume) and then incubated in darkness with shaking (150 rpm). The mycelia from three flasks were harvested every 2 days to determine the mycelia dry weight and measure the HEA content.

### Statistical analysis

The data are expressed as mean ± SD and were analysed by one-way analysis of variance. Significant differences were determined by Duncan’s multiple range tests (*P* < 0.05). Data analyses were completed with SPSS 23.0 (SPSS Inc., Chicago, IL, USA) and OriginPro 8.5 (OriginLab Corporation, Northampton, MA, USA).

## Results

### Identification and verification of HEA production

**Figure 1.**
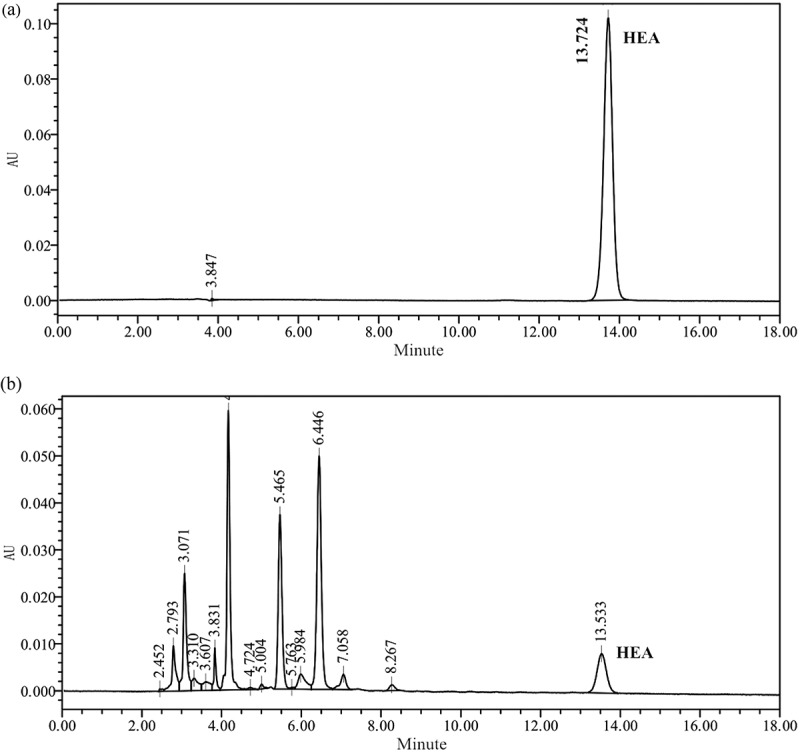
HPLC chromatograms of HEA standard solution and the water extract from mycelium of strain 351. Samples were eluted in the mobile phase, which consisted of acetonitrile: double-distilled H_2_O (0.1% acetic acid) (5:95), for 20 min at 25°C. The flow rate was 1 mL/min and the injection volume was 10 μL. HEA was monitored and quantified at 260 nm.

Of the tested strains, only strain 351 isolated from a *Clitocybe* sp. fruiting body was detected to produce HEA by HPLC spectrogram. The HPLC analysis of the water extract for the strain 351 mycelium revealed a peak at a retention time of 13.533 min (Figure 1(b)), which was consistent with the retention time of the peak for the HEA standard (13.724 min; Figure 1(a)). The strain 351 HEA content was 0.8483 ± 0.0439 mg/g mycelia dry weight.

**Figure 2.**
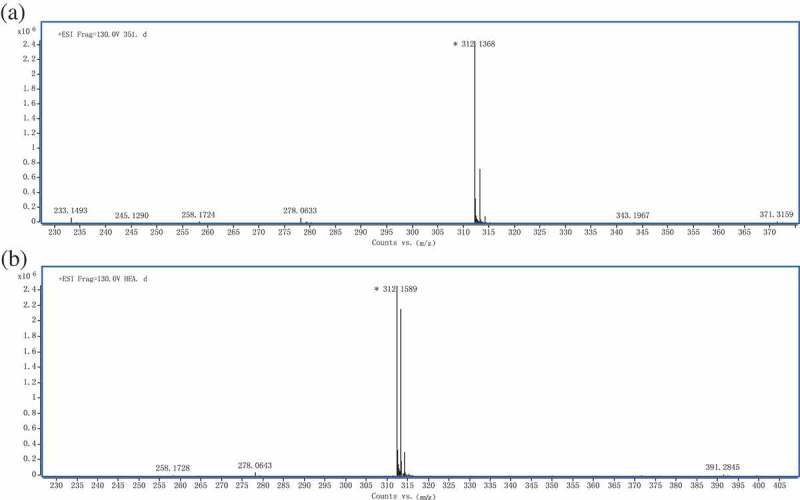
Electrospray mass spectra of HEA isolated from strain 351 and standard solution of HEA. Spectra were scanned over the mass range 80–2000 *m*/*z* at 1.03 spectra/s. The mass fragmentation pattern of the peak at the retention time of 13.533 min is presented with the *m*/*z* spectrum of the peak revealing dominant ions [M + H]^+^ at 312.1 under positive ionisation conditions.

The production of HEA by strain 351 was verified by LC-MS. The mass fragmentation pattern of the peak at the retention time of 13.533 min is presented in Figure 2(a), with the *m*/*z* spectrum of the peak revealing dominant ions [M + H]^+^ at 312.1 under positive ionisation conditions (Figure 2(a)). Thus, the deduced molecular weight was 311, which was similar to the molecular weight of the HEA standard (Figure 2(b)).

### Identification of the HEA-producing strain

**Figure 3.**
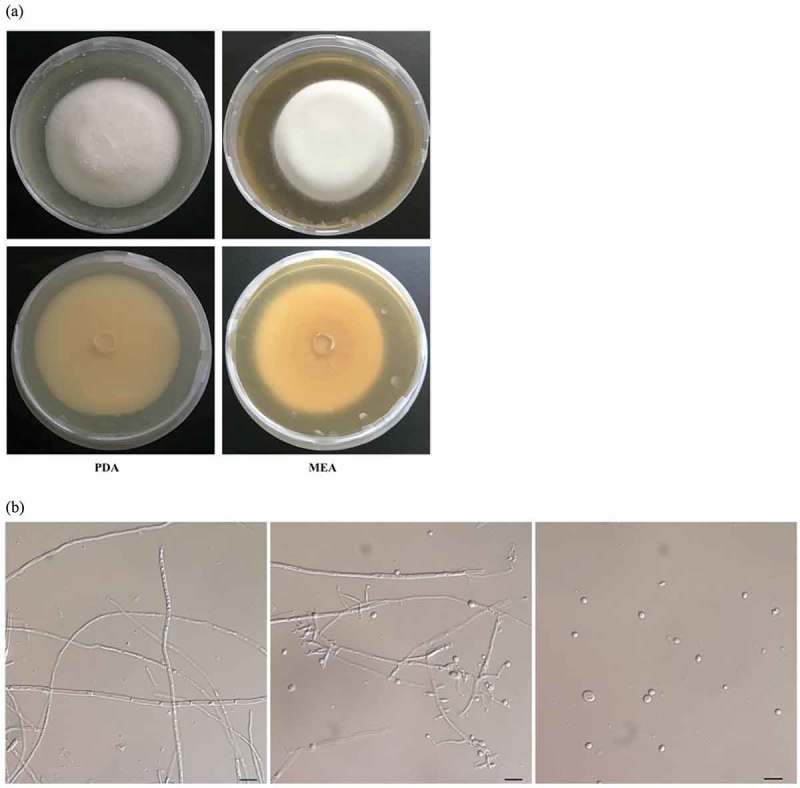
Morphology of the HEA-producing strain 351 (CGMCC 3.18729). (a) Morphology of colonial growth on MEA and PDA medium.The plates in the top show the front view and bottom show the reverse view of the growth after incubation for 10 days at 25°C.(b) Microscopic features of strain 351 taken from MEA showing hypha, conidiophore and conidia (scale bar = 10 μm). Strain 351 was deposited in China General Microbiological Culture Collection Center (CGMCC 3.18729).

The morphological characteristics of the HEA-producing strain 351 colonies were observed on MEA and PDA (Figure 3). The mycelia growth rates were approximately 0.325 mm/day. The strain 351 mycelium generally appeared densely cottony to flocculent, and grew as a yellowish–white, raised and dome-shaped mould on PDA (Figure 3(a)). In contrast, the mycelium on MEA seemed thicker or more swollen. Mycelial fragments were placed on glass slides and a subsequent microscopic observation indicated the hyphae diameter were about 1.2–3.1 μm (Figure 3(b)). The hyphae were branched and formed bottle-like conidiogenous cells with a small neck. The branches were 8.0–23.0 μm long and 1.0–2.0 μm wide (Figure 3(b)). Conidia were oval to subglobose and apiculate, and were 2.0–4.5 μm or (2.0–5.0) × (2.5–4.5) μm (Figure 3(b)). Additionally, strain 351 produced considerably more conidia on MEA than on PDA.

**Figure 4.**
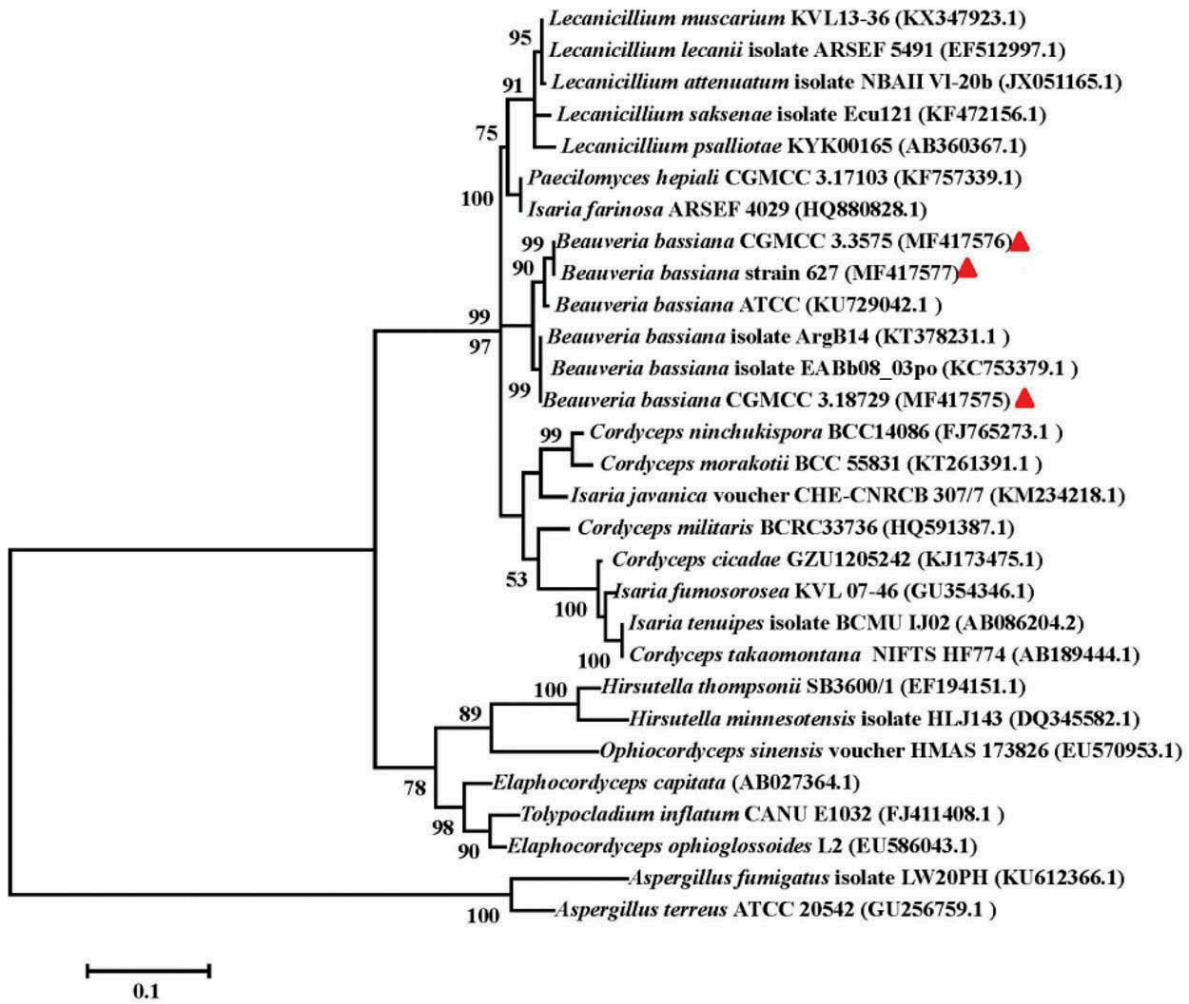
Phylogenetic relationship of CGMCC 3.18729 and the other related species inferred from analysis of ITS rDNA sequences. The numbers at each node represent the percentage of bootstrap support calculated from 1000 replicates. The sequences of strain CGMCC 3.18729, CGMCC 3.3575 and strain 627 indicated with solid triangle were generated in this study, while others were retrieved from GenBank. The ITS sequences of *Aspergillus terreus* (GU256759.1) and *A. fumigatus* (KU612366.1) were used as the out groups.

In addition to morphological characteristics in cultures, a molecular method was used to confirm the strain identity. The ITS1–5.8S–ITS2 nuclear ribosomal DNA sequence was amplified for the cultures. The BLAST searches of the NCBI nucleotide database indicated that ITS sequence of strain 351 exhibited a 100% similarity with *B. bassiana* isolate ArgB14 (KT378231.1). In the phylogenetic analyses (Figure 4), strain 351 formed a clade with *B. bassiana* isolate ArgB14 (KT378231.1) and EABb08_03po (KC753379.1) with 100% bootstrap support. These results confirmed that strain 351 was a *B. bassiana* strain. The fungus was deposited in the collection maintained by the China General Microbiological Culture Collection Center (CGMCC 3.18729).

## Production of HEA in other B. bassiana *isolates*

To ascertain whether the other analysed *B. bassiana* strains also produce HEA, strain 627 and CGMCC 3.3575 were cultured in enriched PDB medium, after which the mycelial extract and culture medium were analysed by HPLC. The HEA content was 0.0089 ± 0.0000 and 0.2673 ± 0.0009 mg/g mycelia dry weight for strain 627 and CGMCC 3.3575, respectively. No HEA was detected in the fermentation broth of all the three tested *B. bassiana* strains. However, adenosine was detected in the mycelia and culture medium for all tested strains (Table 1). The identities of strain 627 and CGMCC 3.3575 were verified by phylogenetic analyses based on the ITS sequences (Figure 4).

**Table 1.** Adenosine and HEA production by different strains of *Beauveria bassiana.*

Strain number		Adenosine (mg/g)^a^	HEA (mg/g)
CGMCC 3.18729 (strain 351)	Mycelium	2.9149 ± 0.0254	0.8483 ± 0.0439
	Broth	1.8027 ± 0.2113	0
CGMCC 3.3575	Mycelium	2.6194 ± 0.0195	0.2673 ± 0.0009
	Broth	1.2325 ± 0.0886	0
Strain 627	Mycelium	2.2000 ± 0.1790	0.0089 ± 0.0000
	Broth	0.2128 ± 0.1071	0

^a^The unit of adenosine content in broth is μg/ml. “g” indicates mycelia dry weight.

### HEA production curve during the liquid culture of strain CGMCC 3.18729

**Figure 5.**
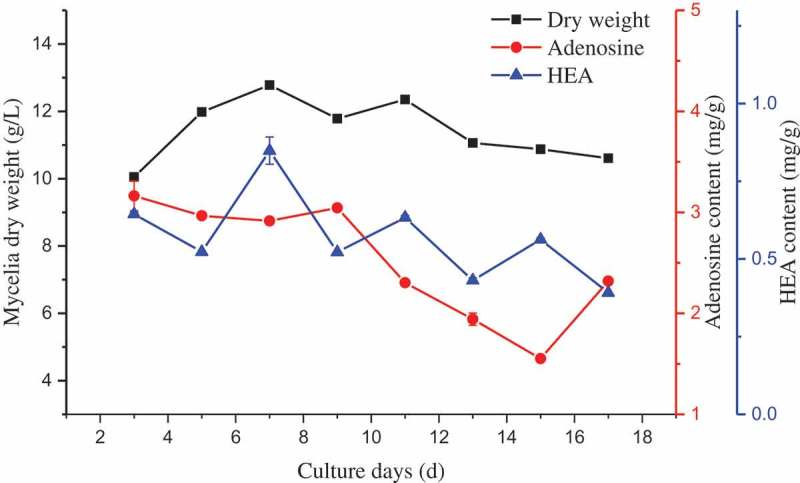
Mycelial biomass and HEA production by strain CGMCC 3.18729 during the liquid culture. The culture was performed in 25°C with 150 rpm for 17 days. The mycelia from three flasks were harvested every 2 days to determine the mycelia dry weight and measure the HEA content.

To study kinetics of HEA production, a typical time course analysis of strain CGMCC 3.18729 grown in a liquid culture was conducted in Erlenmeyer flasks. In submerged cultures, the highest mycelia dry weight (i.e. 12.78 g/L) was accumulated on day 7 after inoculation (Figure 5), while the stationary phase lasted 4 days before shifting to the death phase. An analysis of the kinetics of HEA content revealed a similar time course as that for the biomass production during the culture. The production of HEA was highest on day 7 (i.e. 0.8483 ± 0.0439 mg/g mycelia dry weight) and then decreased slightly by the end of the growth period. Meanwhile, adenosine production decreased over the growth period, with a maximum production of 3.1625 ± 0.1463 mg/g mycelia dry weight on day 3.

## Discussion

HEA was originally isolated from cultured mycelia of *Cordyceps* species. There has been increasing interest in HEA because of its potential medicinal properties, including its ability to inhibit tumour cell proliferation, prevent inflammation, protect kidneys and function as a sedative. Because the production of HEA has been detected only in some *Cordyceps* species, searching for new HEA-producing resources is warranted. In the present study, we observed HEA production in *B. bassiana* under submerged culture conditions. It will provide a new HEA-producing resource and may explore a new application for *B. bassiana* in future.

*B. bassiana* is a ubiquitous fungal pathogen of insects which is used to control a variety of insect pests as a biocontrol agent (Singh et al. 2015). *B. bassiana* is also widely used to treat infantile convulsions, epilepsy, stroke, sore throat and different types of wounds (Madsen et al. 2007). The fungal species produces a plethora of secondary metabolites such as bassiacridin, beauvericin, bassianolide and bassianolone (Singh et al. 2015). Additionally, *C. bassiana*, the teleomorph of *B. bassiana*, produces nucleosides, such as 1,9-dimethylauanine, adenosine, uridine, 3-methyluracil and 1,7-dimethylxanthine (Park et al. 2013; Suh et al. 2017). In this study, we found that *B. bassiana* also produce HEA.

The HEA content in the submerged mycelia of *B. bassiana* strain CGMCC 3.18729 was analysed by HPLC-MS. Moreover, the mycelial HEA contents were examined for two other strains, namely strain 627 and CGMCC 3.3575. The HEA concentration in CGMCC 3.18729 was 0.8483 ± 0.0439 mg/g mycelia dry weight, which is much higher than that reported for *I. cicadae* (Liu et al. 2012) and *C. pruinosa* (Meng et al. 2014). Because HEA is associated with several bioactivities, new applications for *B. bassiana* may be developed in the future.

In this study, we detected HEA in the *B. bassiana* mycelium, but not in the culture medium, unlike adenosine, which was detected either in the mycelia or the culture media. Similarly, HEA has been detected in the mycelia of other *Cordyceps* species, including *I. cicadae, C. pruinosa* and *I. tenuipes*, with very little in the culture media (Li 2011). These results imply that HEA is an intracellular compound.

Some *Cordyceps* species have been used as traditional Chinese medicine for centuries and the manufacturing of *Cordyceps*-derived products represents a major industry in China (Dong et al. 2015, 2016). Adenosine, 3′-deoxyadenosine (cordycepin) and HEA are the major active constituents of *Cordyceps* species. Unlike adenosine, which is widely distributed among *Cordyceps* species (Liu et al. 2012), cordycepin and HEA have been detected in only some *Cordyceps* species. Cordycepin is produced only by *C. militaris* (Meng et al. 2015a), while HEA has been detected in *C. militaris, C. pruinosa, I. tenuipes* and *B. bassiana*. Obviously, there is no correlation between cordycepin and HEA production. Furthermore, the cordycepin biosynthesis pathway, with adenosine as the starting substrate, has been characterised (Xia 2014). We are currently attempting to clarify the HEA biosynthesis pathway.

## Conclusion

In summary, we identified a new HEA-producing fungal strain, which was subsequently identified as *B. bassiana* based on morphological and phylogenetic analyses. The mycelia of two other *B. bassiana* strains from different origins were also detected HEA production. The fungal biomass and kinetics of HEA production exhibited similar trends over the culture period. To the best of our knowledge, this is the first report describing HEA production in a *B. bassiana* strain. This new source of HEA may lead to novel applications for *B. bassiana*.
